# Surgical Management of Multiple Valve Endocarditis Associated with Dialysis Catheter

**DOI:** 10.1155/2016/4072056

**Published:** 2016-11-22

**Authors:** R. Zea-Vera, M. Sanchez, E. Castañeda, L. Soto-Arquiñigo

**Affiliations:** ^1^Universidad Peruana Cayetano Heredia, San Martin de Porres, Lima, Peru; ^2^Cardiovascular Surgery Department, Hospital Cayetano Heredia, San Martin de Porres, Lima, Peru; ^3^Infectious Disease Department, Hospital Cayetano Heredia, San Martin de Porres, Lima, Peru

## Abstract

Endocarditis associated with dialysis catheter is a disease that must be suspected in every patient with hemodialysis who develops fever. Multiple valve disease is a severe complication of endocarditis that needs to be managed in a different way. There is very limited data for treatment and every case must be considered individually. We present a patient with this complication and describe the medical treatment and surgical management. We report the case of a 15-year-old patient with acute renal failure that develops trivalvular endocarditis after the hemodialysis catheter was placed, with multiple positive blood culture for* Staphylococcus aureus*. Transesophageal echocardiography was done and aortic and tricuspid valvular vegetations and mitral insufficiency were reported. Patient was successfully treated by surgery on the three valves, including aortic valve replacement. There is limited data about the appropriate treatment for multiple valvular endocarditis; it is important to consider this complication in the setting of hemodialysis patients that develop endocarditis and, despite the appropriate treatment, have a torpid evolution. In countries where endovenous drug abuse is uncommon, right sided endocarditis is commonly associated with vascular catheters. Aggressive surgical management should be the treatment of choice in these kinds of patients.

## 1. Introduction

Endocarditis is the inflammation of the endocardium, the inner lining of cardiac chambers, and valves; even though there are other causes of endocarditis, infectious etiology is the most common.

First, there is disruption of endocardium through multiple mechanisms, the most common being turbulent flow due to congenital heart diseases in developed countries and rheumatic heart disease in underdeveloped ones; other causes are intracardiac devices and some bacteria with high virulence such as* Staphylococcus aureus* [1].

Then platelets and fibrin deposit within this lesion and a sterile thrombus form. When bacteremia ensues, for example, after dental treatment, there can be bacterial growth within the thrombus due to adhesins [2]. Finally when the vegetation “matures” further fibrin deposit stabilizes the thrombus.

Reported mortality varies from 15 to 30% and has been stable in the past years; nonetheless there has been a variation in risk factors; recently prevalence of endocarditis associated with invasive procedures, higher population age, and diabetes prevalence has increased [3].

In the present case report we present a patient with acute renal injury that develops multiple valve endocarditis (tricuspid, mitral, and aortic) associated with a dialysis catheter, and we also present a literature review highlighting medical treatment and surgical management of infectious endocarditis.

## 2. Case Presentation

A 15-year-old male patient comes to the emergency department with a 4-week history of lower extremity edema and arthralgia of knees and ankles that increases in intensity and restricts deambulation.

Approximately 4 hours before presenting to the emergency room, the patient reports an episode of macroscopic hematuria. At the emergency department, he referred to sleepiness, adequate appetite, and 2-3 kg of weight loss in the last month. The patient denied fever or chills, diarrhea, movement disorders, or other symptoms.

### 2.1. Clinical Findings

Upon physical examination he is found to be alert and fully oriented. Blood pressure was 109/60 mmHg, heart rate was 103 beats per minute and 19 respirations per minute, temperature was 37.4°C, and O_2_ saturation was 95% at room air. Skin paleness and edema of lower extremities were noticed on inspection. The rest of the physical examination was within normal limits.

Laboratory results on admission can be seen on Table 1. Electrocardiogram was within normal limits. Because of abnormal urea, creatinine, and hemoglobin results, hemodialysis was indicated.

### 2.2. Diagnostic Assessment

**Figure 1:**
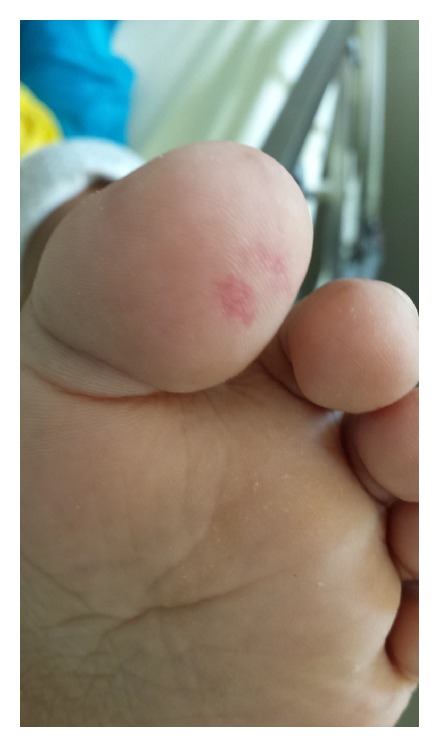
Janeway lesion on patient's left toe.

Within one week after hemodialysis, the patient presented febrile episodes with multiple positive blood culture for methicillin-resistant* Staphylococcus aureus *(MRSA) (Table 2). Upon physical examination, a holosystolic murmur was heard at the tricuspid focus and Janeway lesions in toes where observed (Figure 1). Transesophageal echocardiography reported aortic and tricuspid valvular vegetations as well as mitral insufficiency.

### 2.3. Therapeutic Intervention

Antibiotic therapy with vancomycin for infectious endocarditis was initiated due to the increased risk of coagulase-negative* Staphylococcus*. Later this treatment was maintained when positive cultures for MRSA (Table 3) were available. Because of new onset heart failure and failure to respond to antibiotic therapy, surgical intervention was decided. Before the surgical procedure, the infected catheter was removed and a peritoneal catheter was placed in order to continue renal replacement therapy.

A median sternotomy approach was used; extracorporeal circulation was initiated. After aortic clamping and cardiac arrest with cardioplegia, an incision was made in the right atrium. Vegetations on the right atrium, superior vena cava entrance, inferior vena cava, and tricuspid valve leaflets were removed.

The surgical team proceeded to open the interauricular septum and vegetations on the mitral valve were also removed. After this, the aortic artery was opened and aortic vegetations as well as the valves were removed. The aortic annulus was enlarged with pericardial patch and an aortic valve mechanical prosthesis number 19 (Bicarbon Slimline, Sorin Group, Saluggia, Italy) was implanted.

Finally the aorta and right atrium were closed in standard fashion and aortic clamping and cardiopulmonary bypass were discontinued. Pacemaker wires were placed in the right atrium and right ventricle and the sternum was closed.

### 2.4. Follow-Up and Outcomes

The postoperatory valve culture reported MRSA and the pathology report described, “dense infiltrates with large areas of necrosis and multiple germ colonies in relationship with infectious endocarditis.”

The patient presented a perioperative myocardial infarction. At the time, echocardiogram reported the following: inferior and anteroseptal hypokinesia with ejection fraction of 40%. Captopril adjusted to creatinine clearance and carvedilol 6.25 mg PO twice a day were started to prevent cardiac remodeling.

Later during hospitalization, he also developed spontaneous bacterial peritonitis, suggested by a high level of leukocytes demonstrated in the peritoneal fluid cytology (Table 4), possibly due to peritoneal dialysis catheter infection.

**Figure 2:**
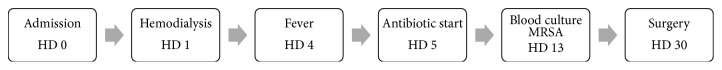
Hospital course.

The catheter was removed and treatment with meropenem 1 g daily was started and continued for 3 weeks. Peritoneal fluid culture was negative (Table 2). The treatment with vancomycin 1 g every 72 hours was stopped after 42 days. Eventually the patient showed good clinical response with remission of disease and was discharged home (Figure 2).

At 6-month follow-up the patient is on oral anticoagulation with an INR within therapeutic ranges. Renal function has improved and he no longer needs dialysis support. From a cardiologic stand-point no new symptoms were reported, EKG remains in normal sinus rhythm with nonspecific ST and T wave changes; lastly follow-up echocardiography has not been performed.

## 3. Discussion

Infectious endocarditis incidence varies from 3 to 10 per 100 000 at risk patients [3], around 60–70% are males [4], and patient age as well as male to female ratio has increased. A recent study in the United States found an increased incidence from 11 to 16 cases per 100 000 at risk people [5]. Mackie et al. also reported increased incidence throughout an 11-year period in Canada [4].

This rise is most likely related to higher predisposition to endocarditis in the general population such as higher population age, higher survival of patients with congenital heart diseases, more patients on hemodialysis, and more intracardiac devices used [6]. Currently healthcare associated endocarditis cases account for 25–30% and are mainly acute [7]. In developing countries the most important predisposing factor is still rheumatic heart disease, making up to one-third of the cases [8].

The percentage of infective endocarditis caused by* Staphylococcus aureus *is increasing; meanwhile Streptococcus viridans and culture negative endocarditis have decreased [9]. Coagulase-negative* Staphylococcus* are part of the normal skin flora but are also the most common cause of early onset prosthetic valve endocarditis; the high association is due to the ability to produce biofilms, high frequency of abscess formation, and increased antibiotic resistance.

In this context the present case illustrates a very difficult clinical situation, where a young patient hospitalized for a renal insufficiency syndrome requiring hemodialysis develops acute multiple valve endocarditis due to MRSA. While hemodialysis is a well-known risk factor for healthcare-related infective endocarditis, the conjunction of the two situations and the potential high mortality risk of endocarditis made endocarditis a prime concern for therapeutic intervention. On its side, the exact etiology of the renal syndrome remained unclear but ultimately improved over time.

### 3.1. Clinical Presentation

Sir William Osler said the following on his, now classic, Gulstonian Lectures: “Few diseases present greater difficulties in the way of diagnosis than malignant endocarditis, difficulties which in many cases are practically insurmountable” [10].

Even though diagnostic tools have certainly improved, the variable clinical presentation requires high suspicion in anyone with sepsis of unknown origin or fever in the presence of risk factors, such as hemodialysis in this case; these patients have an increased calculated incidence up to 308 per 100 000 patient years, and the most common presenting sign is fever [11, 12].

Most common physical findings are fever and a new onset cardiac murmur; also classic findings such as Osler nodes and Janeway lesions are rare (<5%); thereby further laboratory and echocardiographic assessment is necessary.

Laboratory findings are also nonspecific and usually show elevated inflammatory markers and can show normochromic and normocytic anemia. Hematuria can be present in up to 25% of patients [7].

Right sided endocarditis is commonly associated with endovenous drug abuse, but in developing countries such as Peru, endovenous drug abuse is uncommon due to economic and cultural reasons [13]. In this population right sided endocarditis is more commonly associated with dialysis catheters.

### 3.2. Diagnosis

Diagnostic criteria have been developed to help clinician and researchers in decision making; the Duke criteria were first proposed in 1994 and later modified in 2000 [14]. Even though Duke's criteria are helpful for clinicians, they are meant to help classification in clinical research and thereby should never replace clinical judgment [3].

Echocardiography plays a major role in endocarditis diagnosis; currently it is recommended to perform transthoracic echocardiography as an initial imaging study in any patient with suspicion of endocarditis.

Transesophageal echocardiography should be performed when an optimal echocardiographic window is not possible (e.g., previous thoracic or cardiovascular surgery, morbid obesity, and chronic lung disease) or when there is high clinical suspicion even if transthoracic echocardiography is negative [15].

### 3.3. Management

Current American and English guidelines do not recommend antibiotic prophylaxis for all patients undergoing dental procedures, yet the American Heart Association does suggest that it is reasonable to give prophylaxis to those patients with highest risk for endocarditis [16, 17]. Studies on the impact of these new guidelines on endocarditis prevalence and pathogenesis present conflicting results [4, 5].

Management should be by a multidisciplinary team involving cardiology, echocardiography, infectious diseases, and cardiothoracic surgery, and other specialties should be available if required, such as nephrology, spinal surgery, neurology, and neurosurgery [18].

Empirical antibiotic treatment has been proposed by the British Society for Antimicrobial Chemotherapy depending on clinical data, yet involvement of local infectious disease experts is recommended due to different causing agents and resistance patterns [19]. Further treatment should be according to blood culture results [12].

Currently around 50% of patients undergo surgery [7]; indications for surgery are heart failure, uncontrolled infection, and prevention of embolism. It is important to note that surgery is not done in all patients with indications, mostly due to clinical status.

The most common indication for surgery is heart failure, and it's present in approximately 45–60% of cases, timing for surgery in the presence of acute aortic or mitral valve regurgitation varies depending on haemodynamic effects.

Another indication is uncontrolled infection, which refers to persistent infection despite adequate antibiotic treatment; also because infection by highly virulent or resistant organisms is difficult to control medically, it can be managed surgically. Finally, embolism prevention, complicating 20–50% of cases, usually presents within the first 2 weeks of antibiotic treatment and with stroke symptoms; surgery depends on vegetation characteristics [20]. Surgery for recurrent embolism is suggested.

In the present case early surgery (26 days after fever onset) was indicated for both cardiac dysfunction and uncontrolled infection, most probably due to extensive vegetations inhabited by bacterial biofilm-like aggregates (see pathology description).

Early surgery, defined as replacement or repair of a valve in the initial hospitalization, has shown to be associated with better in-hospital survival [21]; this association has also been found on patients in chronic hemodialysis [11]. Nevertheless, early mortality following operation of active endocarditis remains not trivial [22]. It was up to 40% and 50%, respectively, 3 and 6 months after surgery for multiple valve infections versus 10% and 20% for single valve infection.

In this case report we presented a patient with acute renal failure that develops trivalvular endocarditis after placement of hemodialysis catheter and was successfully treated by early surgery on the three valves. In countries where intravenous drug use is uncommon the increased use of hemodialysis catheters should present a new group of high risk patients. Finally, the present case illustrates the good side of the medal as both his cardiac and renal conditions are improving after 6 months of follow-up.

## Figures and Tables

**Table 1:** Laboratory results.

Exam	Result	Laboratory normal values
Glucose	91 mg/dL	75–110 mg/dL

Urea	308.5 mg/dL	19–43 mg/dL

Creatinine	19.4 mg/dL	0.8–1.5 mg/dL

Hemoglobin	5.6 g/dL	11–15 g/dL

Leukocytes	8.740 × 10^3^/mm^3^	5–10 × 10^3^/mm^3^

Platelets	23 200 × 10^3^/mm^3^	150–400 × 10^3^/mm^3^

Sodium	140 mEq/L	135–148 mEq/L

Potassium	5.11 mEq/L	3.5–5.3 mEq/L

Arterial blood gases	pH: 7.00 pCO_2_: 20.5 mmHg pO_2_: 132 mmHg HCO_3_: 4.9	

Urine analysis	>100 RBC per field 3 WBC per field	<3 RBC per field <5 WBC per field

**Table 2:** Culture results.

Hospitalization day (HD)	Culture	Result
11	Blood 1	*Staphylococcus epidermidis*
13	Blood 2	MRSA
17	Blood 3	MRSA
21	Blood 4	MRSA
15	Urine	Negative
34	Peritoneal fluid	Negative

**Table 3:** Antibiogram: MRSA.

Antibiotics	Interpretation
Amikacin	Resistant
Ciprofloxacin	Resistant
Clindamycin	Resistant
Chloramphenicol	Sensible
Erythromycin	Resistant
Gentamicin	Resistant
Levofloxacin	Resistant
Oxacillin	Resistant
Penicillin	Resistant
Rifampicin	Sensible
Tetracycline	Sensible

**Table 4:** Peritoneal fluid cytology.

Cytology	Results	Normal values
Color	Yellow	Yellow
Aspect	Turbid	Transparent
Leukocytes	6900/mm^3^	<500/mm^3^
Polymorphonuclear cells	70%	<25%
Red blood cells	5	0.10–2.00/mm^3^
